# Clinicopathological Features of Gallbladder Papillary Adenocarcinoma

**DOI:** 10.1097/MD.0000000000000131

**Published:** 2014-12-12

**Authors:** Xueshuai Wan, Haohai Zhang, Cuimin Chen, Xiaobo Yang, Anqiang Wang, Chengpei Zhu, Lilan Fu, Ruoyu Miao, Lian He, Huayu Yang, Haitao Zhao, Xinting Sang

**Affiliations:** From the Department of Liver Surgery (XW, HZ, XY, AW, CZ, LF, LH, HY, HZ, XS); Department of pathology, Peking Union Medical College Hospital, Chinese Academy of Medical Sciences and Peking Union Medical College, Beijing, China (CC); and Liver Center and The Transplant Institute, Department of Medicine, Beth Israel Deaconess Medical Center, Harvard Medical School, Boston, MA (RM).

## Abstract

Although patients with gallbladder papillary adenocarcinoma (GBPA) appear to have better prognoses than patients with other pathological subtypes of gallbladder carcinoma (GBC), the clinicopathological features and outcomes of GBPA have not been fully explored. This study therefore analyzed the clinicopathological characteristics and outcomes of GBPA.

This study included 16 patients with GBPA and 101 with gallbladder adenocarcinoma (GBA) not otherwise specified (NOS), all diagnosed pathologically after surgical resection. Clinicopathological and survival data were retrospectively collected and compared.

Fever was significantly more common in GBPA (7/16 vs 10/101; *P* = 0.000). Serum carbohydrate antigen 19-9 level was increased in 1 of 9 patients with GBPA and 39 of 76 with GBA (*P* = 0.022). More patients with GBPA underwent curative resection (15/16 vs 54/101; *P* = 0.009). Pathologically, patients with GBPA were at much earlier tumor (T) (4 in situ, 8 T1; *P* = 0.000) and Tumor, Node, Metastases (TNM) stages (*P* = 0.000). The overall 1-, 3-, and 5-year survival rates were significantly higher in patients with GBPA (100%, 76.9%, and 76.9%, respectively), than in patients with GBA (72.2%, 38.8%, and 31.0%, respectively; *P* = 0.001). Preoperative jaundice (odds ratio 7.69; 95% confidence interval, 1.53–38.76; *P* = 0.013) was a significant prognostic factor in patients with GBA, but was no longer significant when the patients with GBA and GBPA were pooled together.

The clinicopathological features of patients with GBPA differed from those in patients with GBA (not otherwise specified). Pooling of patients may mask prognostic factors in each group.

## INTRODUCTION

Gallbladder carcinoma (GBC), the most common malignancy of the biliary tract, is associated with a dismal prognosis.^1^ The most frequently observed histologic type of GBC is adenocarcinoma, accounting for 80% to 97% of GBCs. Other histopathologic variants include the papillary, mucinous, squamous, and adenosquamous subtypes.^2^ Patients with gallbladder papillary adenocarcinoma (GBPA) have a better prognosis than patients with conventional nonpapillary carcinomas.^3,4^ This has been attributed to the relatively delayed invasion of GBPA into the gallbladder wall, their exophytic growth, and possibly to the early presentation of obstructive symptoms. Interestingly, these characteristics resemble the behavior of intraductal papillary neoplasms of the bile duct and intraductal papillary mucinous neoplasms of the pancreas, considered the counterparts of GBPA in the bile duct and pancreas, respectively.^5–7^ To better characterize the clinicopathological features of GBPA, this study compared the features of GBPA with those of gallbladder adenocarcinoma (GBA) not otherwise specified (NOS).

## METHODS

Of the patients who underwent surgical resection for gallbladder diseases at Peking Union Medical College Hospital, Beijing, China, between May 1990 and December 2013, 16 were pathologically diagnosed with GBPA and 101 with GBA. All participants provided written informed consent, and all study procedures were approved by the Peking Union Medical College Hospital Ethics Committee.

The clinicopathological characteristics of the included patients were retrospectively reviewed, including sex; age; symptoms; physical examination results; presence of gallstones, smoking; alcohol drinking; diabetes mellitus; hypertension; liver function tests; serum concentrations of the tumor markers carcinoembryonic antigen (CEA) and carbohydrate antigen 19–9 (CA19-9); results on ultrasound (US) and computed tomography (CT); type of surgery; tumor location and maximum size; histologic differentiation; TNM stage; date of surgery; and date of death or last follow-up.

History and clinical data were obtained from medical records. Liver function and serum tumor marker assays were considered positive when concentrations exceeded the higher limit of the normal range. The presence of tumor or wall thickening on US and CT images was assessed by 2 independent radiologists. Type of surgery type was classified as curative or noncurative resection. Curative (R0) resection was defined as nonresidual tumor, whereas the presence of microscopic (R1 resection) or macroscopic (R2 resection) residual tumor was considered noncurative. Pathological diagnoses were confirmed by 2 pathologists. Overall survival (OS) was defined as the time interval from the date of surgery to the date of death. Follow-up data were obtained from outpatient clinic visits, phone calls, and questionnaires submitted by mail.

### Statistical Analysis

Categorical variables were reported as number and compared using *χ*^2^ tests. Continuous variables were reported as mean ± standard deviation and compared using Mann–Whitney *U* tests. OS was analyzed using the Kaplan–Meier method and compared using log-rank tests. Cox regression analysis was performed to determine factors prognostic of survival in each group. All potential prognostic factors on univariate analyses were entered into the multivariable Cox models. A *P* value <0.05 was considered statistically significant. All statistical analyses were performed using The Statistical Package for the Social Sciences for Windows (SPSS Inc, Chicago, IL).

## RESULTS

### Clinical Characteristics

The mean ages of patients with GBPA and GBA were similar, 66.9 ± 12.9 years (median, 71 years; range, 36–89 years) and 65.1 ± 9.8 years (median, 65 years; range, 29–85 years), respectively (*P* = 0.347), and the female:male ratios of the 2 groups were 3:1 and 1.7:1, respectively (*P* = 0.365). Abdominal pain was the most common presenting symptom in the 2 groups (10/16 vs 80/101; *P* = 0.141), but fever was significantly more frequent in patients with GBPA than GBA (7/16 vs 10/101; *P* = 0.000). Among patients with fever, 3 of 7 in the GBPA group and 5 of 10 in the GBA group also had cholecystolithiasis (*P* = 0.772). Physical examination showed that right upper quadrant mass or tenderness was relatively rare in both groups. The percentages of patients with cholecystolithiasis (6/16 vs 59/101; *P* = 0.118), smoking, alcohol drinking, diabetes mellitus, and hypertension, and the results of liver function tests, were similar in the GBPA and GBA groups. Increased serum CA19-9 concentration was observed in 1 of 9 patients with GBPA and in 39/ of 76 patients with GBA (*P* = 0.022); however, the percentages of patients with higher CEA levels did not differ significantly (2/10 vs 22/75; *P* = 0.538).

Fourteen of 16 patients (87.5%) with GBPA and 73 of 97 (75.3%) with GBA who underwent US were suspected of having gallbladder carcinoma. CT scans were considered positive in 10 of 12 (83.3%) and in 64 of 75 (85.3%) patients, respectively. Both GBPA and GBA lesions were found to be polymorphic masses with a rich blood supply; however, wall thickening was more frequently detected on US or CT in patients with GBA than with GBPA. Of the 99 patients with GBA and the 16 with GBPA who underwent at least 1 US or CT examination, 52 (52.5%) and 3 (18.8%), respectively, showed evidence of gallbladder wall thickening, with or without a detectable tumor (*P* = 0.012). Demographic data and clinical characteristics are summarized in Table 1.

**TABLE 1:**
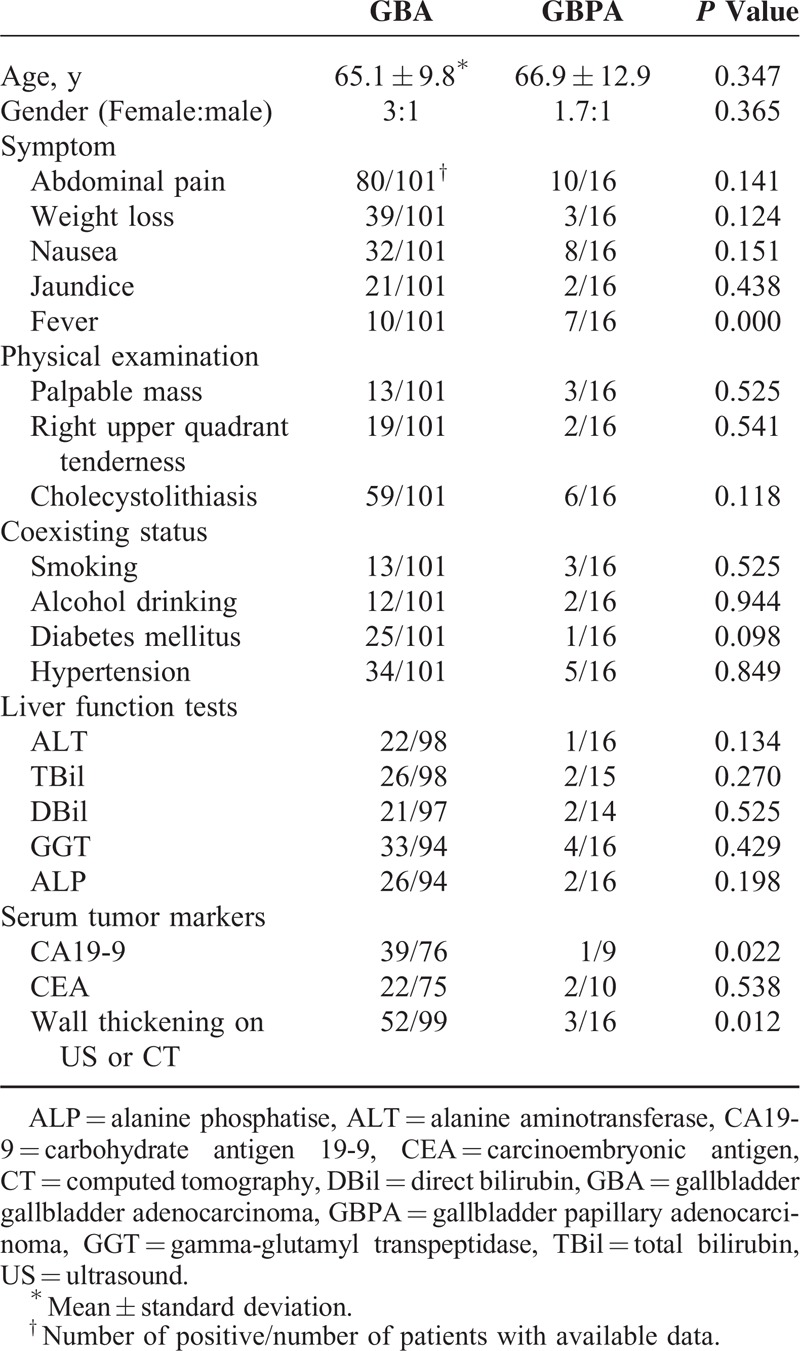
Demographic Data and Clinical Characteristics of Gallbladder Papillary Adenocarcinoma and Gallbladder Adenocarcinoma

### Surgical and Pathological Characteristics

Of the 16 patients with GBPA, 15 (93.8%) underwent R0 resection and 1 (6.3%) underwent R2 resection. Of the 101 patients with GBA, however, 54 (53.5%) underwent R0, 21 (20.8%) underwent R1, and 26 (25.7%) underwent R2 resections (*P* = 0.009; Table 2).

**TABLE 2:**
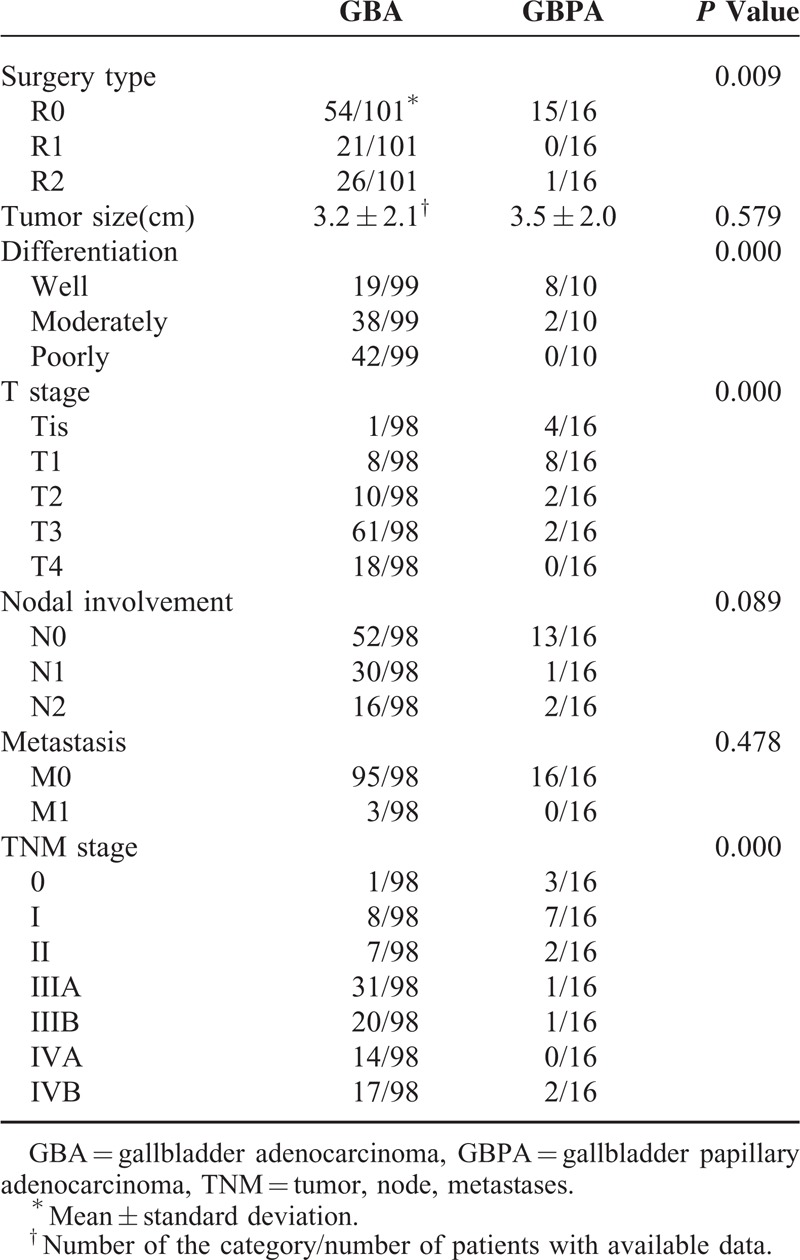
Surgical and Pathological Characteristics of Gallbladder Papillary Adenocarcinoma and Gallbladder Adenocarcinoma

**FIGURE 1:**
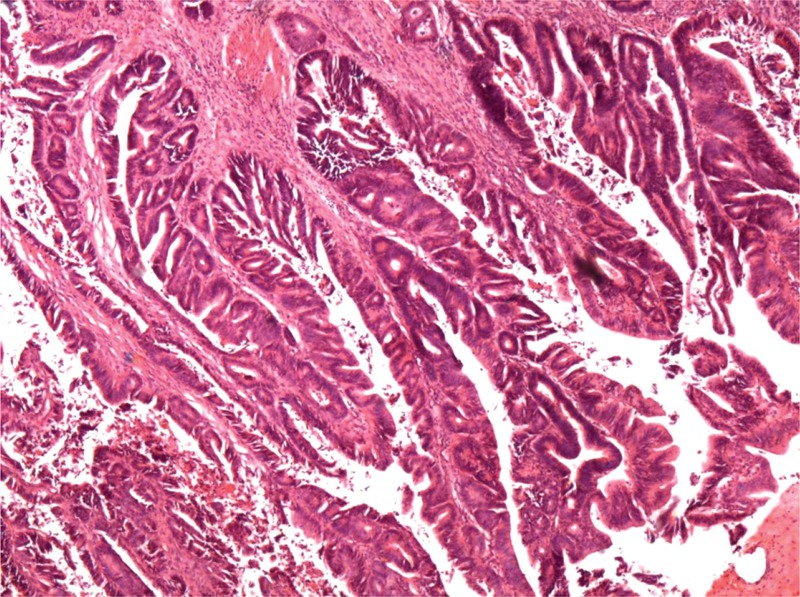
Histological appearance of a gallbladder papillary adenocarcinoma. Microscopically, these tumors present with papillary proliferation of epithelial cells and delicate fibrovascular stalks (hematoxylin and eosin staining × 40).

Pathological examination of surgical specimens showed that GBPA lesions appeared macroscopically as sessile polypoid nodules or as cauliflower-like masses projecting into the lumen of the gallbladder. The mean tumor size of GBPAs was 3.5 ± 2.0 cm, similar to that of GBAs (3.2 ± 2.1 cm; *P* = 0.579). Histologically, GBPAs showed papillary proliferation of epithelial cells with delicate fibrovascular stalks (Figure 1). Most of these lesions were moderately to poorly differentiated, whereas most GBPAs were well differentiated (*P* = 0.000). Tumors in all 16 patients with GBPA and in 98 with GBA were staged according to the TNM staging system of the 7^th^ AJCC (Table 2). GBPA lesions were at much earlier T stages (4 in situ, 8 T1), whereas the majority of GBA lesions were at T3 and T4 stages (*P* = 0.000). Although between-group differences in nodal involvement and distance metastasis were not observed, TNM stages differed for GBPA and GBA lesions (*P* = 0.000; Table 2).

### Overall Survival of GBPA and GBA

**FIGURE 2:**
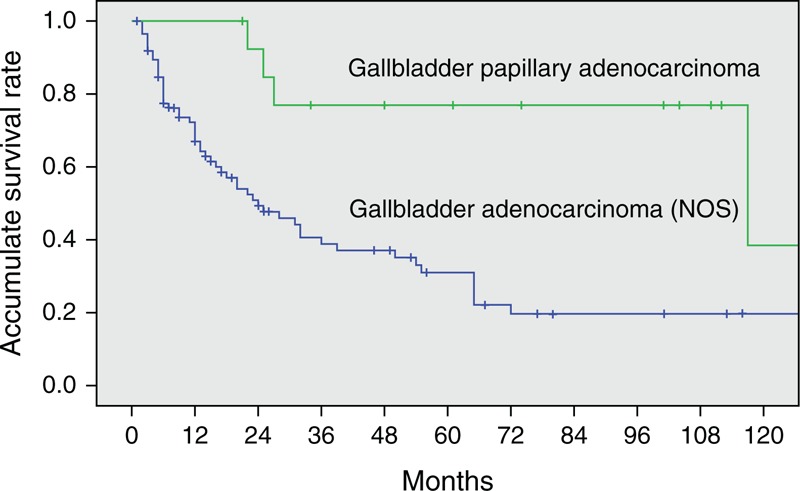
Kaplan–Meier analysis of cumulative overall survival rates in patients with gallbladder papillary adenocarcinoma and gallbladder adenocarcinoma. Overall survival was significantly longer in the former group (*P* = 0.001).

**FIGURE 3:**
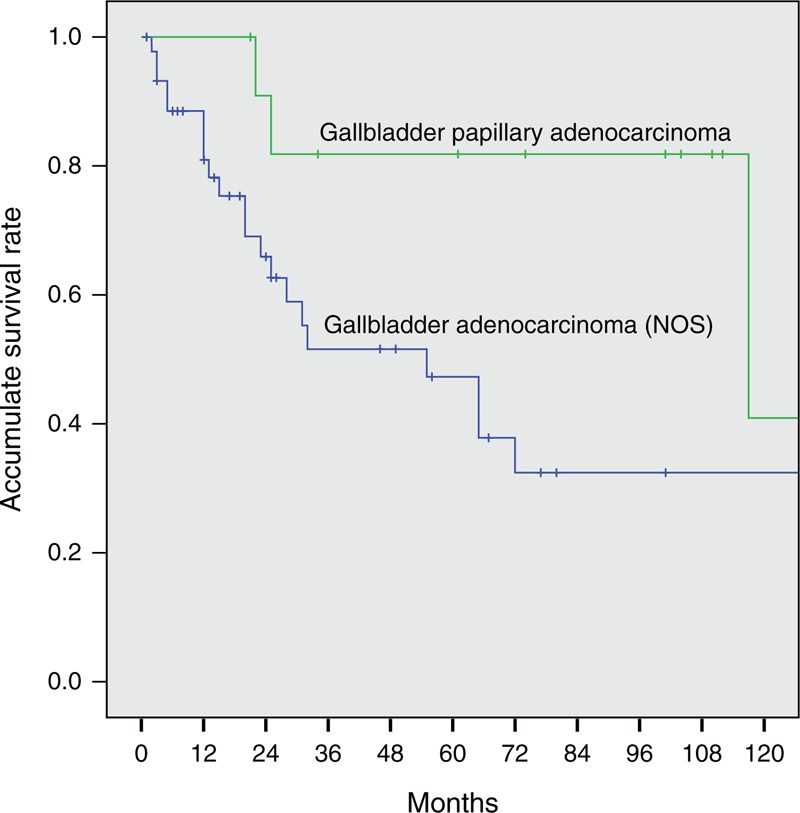
Kaplan–Meier analysis of cumulative overall survival rates in patients with stage N0 gallbladder papillary adenocarcinoma and stage N0 gallbladder adenocarcinoma. Overall survival was significantly longer in the former group (*P* = 0.021).

Three patients with GBA who died of postoperative complications within 1 month during hospitalization were excluded. At the time of analysis, 8 of 16 patients (50.0%) with GBPA and 24 of 98 (24.5%) with GBA were alive. At a median follow-up of 61 months (range, 21–235 months) for patients with GBPA and 14 months (range, 1–138 months) for patients with GBA, the median OS was significantly longer for patients with GBPA (117.0 months; 95% confidence interval [CI], 0–244.6 months) than for patients with GBA (24.0 months; 95% CI, 12.4–35.6 months; *P* = 0.001). The 1-, 3-, 5-, and 10-year OS rates were 100%, 76.9%, 76.9%, and 38.5%, respectively, in patients with GBPA and 72.2%, 38.8%, 31.0%, and 19.7%, respectively, in patients with GBA (Figure 2). Although we also compared OS by tumor stages in patients with GBPA and GBA, the number of patients in the GBPA group at each T or TNM stage was too small to detect statistical differences. However, median OS differed significantly in patients with GBPA and GBA without node involvement (stage N0), being 117.0 months (95% CI, 0–247.1 months) and 55.0 months (95% CI, 20.2–89.8 months), respectively (*P* = 0.021, Figure 3). In addition, T stage (*P* = 0.000) and wall thickening on imaging (*P* = 0.002) differed significantly in patients with stage N0 GBPA and GBA.

### Prognostic Factors in Patients with GBA and GBA Plus GBPA

**FIGURE 4:**
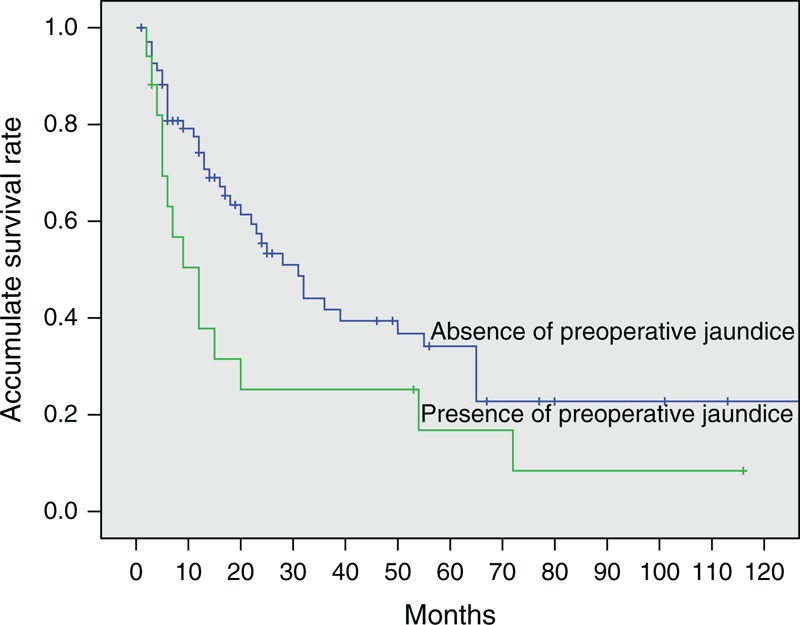
Kaplan–Meier analysis of cumulative overall survival rates in patients with gallbladder adenocarcinoma with and without preoperative jaundice (*P* = 0.001).

Because of the small number of patients with GBPA, we could not determine any factors prognostic for survival in this group. We therefore analyzed prognostic factors in patients with GBA and in those with both GBPA and GBA to determine whether there were any differences in survival predictors between the GBPA and GBA groups. Univariate analysis showed that jaundice; nausea; concentrations of alanine aminotransferase, total bilirubin, direct bilirubin, gamma-glutamyl transpeptidase, alanine phosphatase, CEA, and CA19-9; type of surgery; and T, N, and TNM stages were associated with OS in patients with GBA. When all of these potential prognostic factors above were entered into multivariate Cox models, we found that preoperative jaundice (odds ratio [OR] 7.69; 95% CI, 1.53–38.76; *P* = 0.013; Figure 4), T stage, and nodal involvement (N stage) were significantly prognostic of survival in the GBA group (Table 3). However, when we pooled patients with GBA and GBPA together, we found that only T stage and N stage were significant predictors of survival on multivariate analysis (Table 4).

**TABLE 3:**
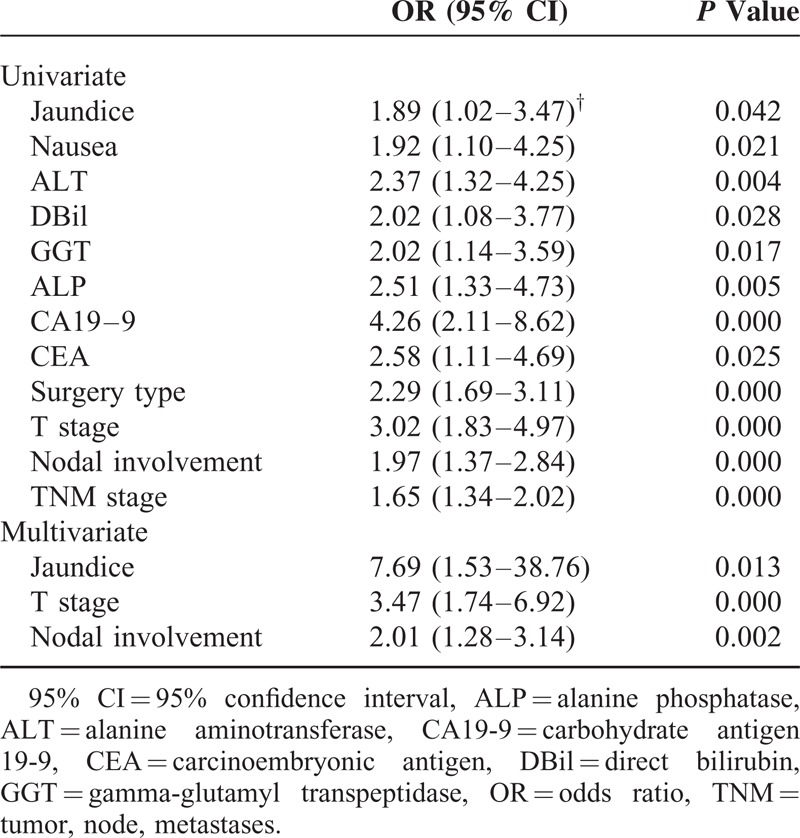
Results of Univariate and Multivariate Analyses for Gallbladder Adenocarcinoma

**TABLE 4:**
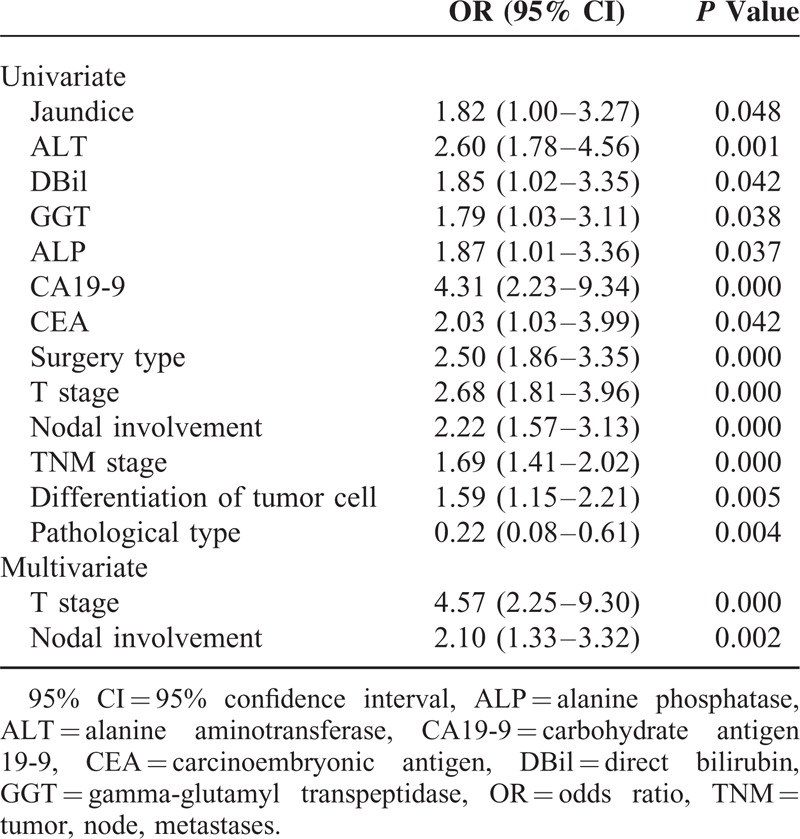
Results of Univariate and Multivariate Analyses for Gallbladder Papillary Adenocarcinoma and Gallbladder Adenocarcinoma Together

## DISCUSSION

Although GBC is the most common cancer of the biliary tract, it remains relatively uncommon and is therefore treated as a single entity. However, GBC includes several pathological subtypes, with adenocarcinoma being the most common. However, papillary adenocarcinoma accounts for nearly 5% of all malignant gallbladder tumors.^8,9^ Although evidence has suggested that the clinical outcomes of patients with GBPA were unexpectedly favorable,^3,4,10^ the unique characteristics of these tumors and their exact clinical outcomes are still largely unknown.

Demographically, we found that GBPA, like GBA, was most common in women in their 70 s. Other similarities between patients with GBA and GBPA included the rates of co-occurrence of cholecystolithiasis and other underlying factors. In contrast, fever, which was uncommon in patients with GBA, was present in nearly half of the patients with GBPA. This was likely not due to cholecystolithiasis, as its prevalence was similar in patients with GBPA and GBA. Rather, fever may result from the exophytic growth pattern of GBPA. Friable tumor emboli can easily detach from their origins, leading to acute obstruction of the cystic duct with subsequent onset of fever.^3,11^ Similarly, patients with acute cholecystitis were reported to have early-stage tumors and better survival outcomes than those who did not.^12^

Elevated serum CA19-9 concentration has been considered helpful in the diagnosis of gallbladder cancer,^13,14^ but may also predict poorer OS after surgical resection.^15,16^ In our study, univariate analysis showed that elevated CA 19-9 was associated with OS in patients with GBA, and that this effect remained after pooling of patients with GBA and GBPA. Nevertheless, elevated CA19-9 was detected in a significantly higher percentage of patients with GBA than with GBPA, suggesting that CA19-9 may be a diagnostic and prognostic factor only in patients with GBA, not in those with GBPA.

On US and CT, both GBPA and GBA can be identified by the presence of a polymorphic mass protruding into the lumen or completely filling it, or by focal or diffuse thickening of the gallbladder wall.^12^ Gallbladder wall thickening likely reflects infiltration by the tumor.^17^ In the present study, wall thickening was more frequently detected on US or CT in patients with GBA than with GBPA. This finding may suggest that GBA can infiltrate the gallbladder wall easier than GBPA.

The poor prognosis of patients with GBC has been attributed to its aggressive biology and advanced tumor stage at presentation.^12^ OS was found to be significantly better in patients who underwent curative than palliative treatment.^18^ However, the resection rate was low, ranging from 22% to 38% in previous series.^19–21^ Moreover, R0 resection can be achieved only in about 50% of patients even with extended resection.^19,22–24^ Of our 101 patients with GBA, 54 underwent R0 resection, similar to the percentage in previous studies, and significantly lower than the percentage of patients with GBPA. The high proportion of patients with GBPA who achieved R0 resection may reflect the relatively earlier stage at detection of these tumors and their less invasive nature. This may be supported by our finding that most GBPAs presented at higher grades of differentiation, although tumor size and age of onset were similar to those of GBA. In addition, GBPAs presented at earlier T and TNM stages. The majority of GBA lesions were classified as T3 (n = 61) or T4 (n = 18), with only 1 of 98 diagnosed as a carcinoma in situ. In contrast, 4 of the 16 GBPAs were classified as carcinomas in situ, with all 4 carcinomas located within papillary adenomas of the gallbladder. This was consistent with the hypothesis that papillary adenocarcinomas may originate from papillary adenomas.^25^ However, a study of gallbladder adenomas reached the opposite conclusion.^26^ Therefore, it may be more suitable and convincing to study different pathologic types of GBC separately.

In the present study, the 1-, 3-, and 5-year accumulative OS rates of patients with GBA were 72.2%, 38.8%, and 31.0%, respectively, consistent with findings in patients with GBC who underwent surgical resection with curative intent.^19,20,27^ In comparison, the OS rates were significantly higher in patients with GBPA than GBA, with 1-, 3-, and 5-year accumulative OS rates of 100%, 76.9%, and 76.9%, respectively. When assessed by stage, we found that OS was significantly better in patients with stage N0 GBPA than in patients with stage N0 GBA. Therefore, although could not analyze patients at each T stage because of the small numbers of GBPA patients with each, our findings suggest that the better prognosis observed in patients with GBPA was due to the reduced ability of these tumors to infiltrate the gallbladder wall and to the possible earlier occurrence of obstructive symptoms associated with GBPA lesions. However, the oncogenic mechanisms underlying these characteristics of GBPA lesions remain unclear, indicating a need for further investigations.

Identification of potential prognostic factors in patients with GBC can help select appropriate surgical strategies and predict life expectancy. Univariate and multivariate analyses showed that T and N stages were independent prognostic factors in the 117 patients with both GBA and GBPA, with both of these factors, plus the occurrence of preoperative jaundice, being prognostic in patients with GBA. Although each of these factors had been identified previously,^19,27–30^ preoperative jaundice, the most powerful predictor in patients with GBA, with an OR of 7.69, was likely masked by the inclusion of patients with GBPA. Jaundice in patients with gallbladder carcinoma is generally considered a consequence of tumor involvement of the extrahepatic bile duct, indicating advanced disease and poor prognosis. This study confirmed that jaundice predicted a poorer prognosis for patients with GBA. Interestingly, it lost significance when patients with GBA and GBPA were pooled, although the incidence of jaundice was similar in the GBA and GBPA groups (*P* = 0.438). This discrepancy suggested that the causes of jaundice differed in patients with GBPA and GBA. Since GBPAs showed less-invasive behavior, jaundice in these patients may have resulted from obstruction by the primary exophytic tumor or detached tumor emboli rather than by locally advanced disease. Although we were unable to identify any factors prognostic of survival in patients with GBPA, due to the small number of patients with these tumors, the differences we observed between patients with both GBPA and GBA and those with GBA alone suggest that GBPA may have unique prognostic factors and should not be confused with GBA.

Several malignant papillary neoplasms of the pancreatobiliary system show a relatively indolent clinical course, with slow pathological transformation of any type, from low-grade dysplasia to invasive carcinoma. Papillary neoplasms of the gallbladder, an inherent part of the pancreatobiliary system, have features similar to those of intraductal papillary mucinous neoplasms of the pancreas and intraductal papillary neoplasms of the bile duct. Less is known about gallbladder papillary neoplasms than about these other two entities, although this study provided additional knowledge about this uncommon disease.

This study had several limitations. First, the number of patients was relatively small, limiting the statistical power of this study. Second, the effects of postoperative chemotherapy and radiotherapy on prognosis were not considered. However, these adjuvant therapies have limited benefits in patients with gallbladder cancer and were therefore unlikely to confound the difference in OS between the GBPA and GBA groups. Additional studies, including larger numbers of patients and focusing on their immunohistochemical and genetic characteristics, are needed.

In conclusion, patients with GBPA presented more frequently with fever, were less likely to have elevated serum CA19-9 concentrations, and were less likely to show wall thickening on US or CT imaging than patients with GBA. Moreover, patients with GBPA tended to undergo R0 resection more frequently and at earlier pathologic TNM stages than patients with GBA. In addition, OS was significantly longer in patients with GBPA than GBA. However, factors predictive of patient survival could be masked by pooling patients with GBPA and GBA. Taken together, these findings suggest that GBPA and GBA be considered separate entities, especially when assessing their postoperative prognosis.
